# Adiponectin modulates ventral tegmental area dopamine neuron activity and anxiety-related behavior through AdipoR1

**DOI:** 10.1038/s41380-018-0102-9

**Published:** 2018-07-09

**Authors:** Fengjiao Sun, Yun Lei, Jingjing You, Chen Li, Linshan Sun, Jacob Garza, Di Zhang, Ming Guo, Phillip E. Scherer, Daniel Lodge, Xin-Yun Lu

**Affiliations:** 1 Department of Pharmacology, The University of Texas Health Science Center at San Antonio, San Antonio, TX USA; 2 Institute for Metabolic & Neuropsychiatric Disorders, Binzhou Medical University Hospital, Shandong, China; 3 Department of Neuroscience & Regenerative Medicine, Medical College of Georgia, Augusta University, Augusta, GA USA; 4 Touchstone Diabetes Center, Department of Internal Medicine, University of Texas Southwestern Medical Center, Dallas, TX USA

**Keywords:** Neuroscience, Psychiatric disorders

## Abstract

Adiponectin, a metabolic hormone secreted by adipocytes, can cross the blood–brain barrier to act on neurons in different brain regions, including those involved in stress-related disorders. Here we show that dopamine neurons in the ventral tegmental area (VTA) express adiponectin receptor 1 (AdipoR1). Intra-VTA infusion of adiponectin or the adiponectin mimetic AdipoRon in wild-type mice decreases basal dopamine neuron population activity and firing rate and reverses the restraint stress-induced increase in dopamine neuron activity and anxiety behavior. Adiponectin haploinsufficiency leads to increased dopamine neuron firing and anxiety behavior under basal conditions. Ablation of AdipoR1 specifically from dopamine neurons enhances neuronal and anxiogenic responses to restraint stress. The effects of intra-VTA infusion of adiponectin on neuronal activity and behavior were abolished in mice lacking AdipoR1 in dopamine neurons. These observations indicate that adiponectin can directly modulate VTA dopamine neuron activity and anxiety behavior, and that AdipoR1 is required for adiponectin-induced inhibition of dopamine neurons and anxiolytic effects. These results strengthen the idea of adiponectin as a key biological factor that links metabolic syndrome and emotional disorders.

## Introduction

The mesolimbic dopamine system, originating from dopamine neurons in the ventral tegmental area (VTA), is a key neural substrate for reward processing and emotional responses [1]. Dysfunction of this system has been linked to a number of psychiatric disorders including anxiety and depression [1–4]. While dopamine neurons are well known for their excitatory responses to rewards, it is becoming increasingly clear that the activity of dopamine neurons is also associated with aversive, stressful stimuli [3–8]. The activity of dopamine neurons is modulated not only by synaptic inputs but also by peripheral hormones [9–11]. The central and peripheral signals act in concert to determine the neuron’s response to both rewarding and aversive/stressful stimuli.

Research suggests a bidirectional association between metabolic syndrome and anxiety [12, 13]. These two conditions may share a common biological basis. It has been shown that midbrain dopamine neurons respond directly to a distinct set of metabolic hormones and relay signals from periphery to mesolimbic neural circuits [9–11, 14]. These studies have mainly focused on the functions of metabolic hormones in food rewards and energy metabolism. Given the critical role of the dopaminergic system in stress responses and emotional behavior [3–8], it is hypothesized that metabolic hormones may modulate stress and emotional processing via modulating dopamine neuron activity. Indeed, the adipocyte-derived hormone leptin was found to regulate VTA dopamine neuron firing and anxiety-related behavior [10, 14, 15]. However, whether similar effects extend to other adipocyte hormones is yet unknown.

Adiponectin, the most abundant circulating adipokine, is secreted exclusively by adipocytes [16, 17]. In contrast to increased levels of leptin, adiponectin levels are reduced in individuals with obesity [18–22]. The anti-diabetic metabolic effects of adponectin have been well characterized [23, 24]. Evidence suggests that adiponectin is able to cross the blood–brain barrier and act on specific neuronal populations through its receptors, AdipoR1 and AdipoR2 [25–28]. These two receptor subtypes have distinct distribution patterns [29], different binding affinity for adiponectin and signaling preferences [24, 28, 30]. However, the precise physiological functions of AdipoR1 and AdipoR2 in the central nervous system remain to be elucidated. While AdipoR1 is widely expressed in the brain, AdipoR2 expression is restricted to only a few brain regions, including the hippocampus and hypothalamus [29]. We recently reported that AdipoR2 regulates neuronal excitability of hippocampal granule neurons and contextual fear in a mouse model of post-traumatic stress disorder [31]. In this study, we revealed that VTA neurons express AdipoR1 mRNA, suggesting that the VTA is a target for adiponectin action. We further investigated the effects of adiponectin in modulating VTA dopamine neuron firing activity and anxiety-like behavior under both basal and stress conditions. To test the hypothesis that AdipoR1 in dopamine neurons is necessary for the neuronal and behavioral effects of adiponectin, we generated mice lacking AdipoR1 in dopaminergic neurons. Our results indicate that adiponectin decreases the activity of VTA dopamine neurons and induces anxiolytic responses through direct activation of AdipoR1.

## Materials and methods

### Animals

C57BL/6J, DAT-IRES-cre and Ai14 mice were purchased from Jackson Laboratory (Bar Harbor, ME, USA). Heterozygous adiponectin gene knockout (Adipo^+/-^) mice were originally obtained from Dr. Philipp Scherer and maintained on a C57BL/6J genetic background [32]. To confirm the specificity of DAT-IRES-cre-mediated recombination in dopaminergic neurons, DAT-IRES-cre mice were crossed with the Ai14 tdTomato reporter line to obtain DAT-IRES-cre, Ai14 mice with tdTomato fluorescence in Cre-expressing cells, which was used to determine the colocalization of tdTomato with a dopamine neuron marker. To generate conditional knockout mice lacking adiponectin receptor 1 (AdipoR1) in dopamine neurons, AdipoR1^flox/flox^ mice, in which exon 2 is floxed [31], were crossed with DAT-IRES-cre mice. The AdipoR1^flox/+^, DAT-IRES-cre offspring were backcrossed with AdipoR1^flox/flox^ mice to generate AdipoR1^flox/flox^, DAT-IRES-cre (AdipoR1^flox/flox^/DAT^IREScre^) and AdipoR1^flox/flox^ littermate controls (Fig. 4a). The PCR primers used for genotyping were as follows: Adiponectin, forward-5′-GGACCCCTGAACTTGCTTCAC-3′, reverse-5′-CACCCACAGTAATTCCATGGG-3′, Neo-reverse-5′-GAATGGGCTGACCGCTTCCTCGTG-3′ [32]; DAT^IREScre^, forward-5′-TGGCTGTTGGTGTAAAGTGG-3′, reverse-5′-CCAAAAGACGGCAATATGGT-3′, and AdipoR1 WT and flox, forward-5′-CCCTGGGGATAGTTCTGGAT-3′, reverse-5′-TTACTCACTGGGCCCTGCTTG-3′. All mice were housed in groups of five per cage under a 12-h light/12-h dark cycle (lights on at 7:00 am) with *ad libitum* access to food and water. For the experiments, male mice at an age between 8 and 12 weeks were used and all animal procedures were approved by the Institutional Animal Care and Use Committee of University of Texas Health Science Center at San Antonio and Binzhou Medical University Hospital.

### RNA extraction and reverse transcriptase PCR

Mice were decapitated and their brains were removed immediately. Coronal brain slices (1 mm) were cut  using a mouse brain matrix/slicer (Braintree Sci., Inc., MA). The VTA area from each slice was then punched out using a well-polished tissue punch needle. VTA punches were homogenized and total RNA was extracted with a total RNA rapid extraction kit. HiScript II QRT SuperMix (Vazyme) was used to generate cDNA according to the following procedure: 300 ng of total RNA and 4 × *g*DNA wiper mix were incubated at 42 °C for 2 min to remove genome contamination, then 5 × HiScript II QRT SuperMix was added to the reaction mixture and incubated at 25 °C for 10 min, 50 °C for 30 min, and 85 °C for 5 min. The resulting cDNA was used for real-time PCR detection using a StepOnePlus real-time PCR system (Applied Biosystems, Waltham, MA, USA). The primer sequences used to amplify each product were as follows: AdipoR1 exon 2: forward-5′-CCCGTATCCACCAGACACCGG-3′; reverse-5′-GGCAATGGGGCTCCTTCTGG-3′ [31], mouse β-actin: forward-5′-GATCATTGCTCCTCCTGAGC-3′, reverse-5′-ACTCCTGCTTGCTGATCCAC-3′ [33]. The ΔΔCT method was used to obtain relative fold-change of target gene expression normalized by the housekeeping gene β-actin compared with control samples.

### Drugs

Recombinant mouse full-length adiponectin (R&D Systems, Minneapolis, MN, USA) was dissolved in artificial cerebrospinal fluid (aCSF, 137 mM NaCl, 2.7 mM KCl, 0.5 mM MgCl_2_·6H_2_O, 0.9 mM CaCl_2_·2H_2_O, 1.5 mM KH_2_PO_4_, and 8.1 mM Na_2_HPO_4_). AdipoRon (AdipoGen, San Diego, CA, USA) was dissolved in dimethyl sulfoxide as a stock solution (30 mM), which was diluted with aCSF for intra-VTA injection. All mice were randomly assigned to different treatment groups.

### Cannula implantation and microinjection

For intra-VTA microinjections, mice were anesthetized with an intramuscular injection of a cocktail containing ketamine 60 mg/ml, acepromazine 1 mg/ml, and xylazine 8 mg/ml (0.1 ml/kg, intramuscular) and mounted onto a stereotaxic frame. The skull surface was first coated with Kerr phosphoric acid gel etchant (Kerr USA). As described elsewhere [15, 29], after the bilateral guide cannula was inserted into the VTA (coordinates: 3.2 mm posterior to the bregma, 0.4 mm lateral to the midline, and 3.8 mm ventral to bregma), Kerr Prime was applied onto the skull and cannula surface. Then adhesive was brushed on top of the primer layer and light cured for 45 s with the VALO curing light (Ultradent Products). Finally, the dental cement was used to fill the area around the cannula and a dummy cannula was inserted into the guide cannula to maintain the cannula patency. Following surgery, animals were individually housed, handled daily and allowed to recover for 7 days. Microinjections were performed on conscious, unrestrained and freely moving mice in their home cages. On the experimental day, a 33-G stainless-steel injector connected to a 5-μl syringe was inserted into the guide cannula and extended 1 mm beyond the tip. AdipoRon (2 nmol/μl) or vehicle was infused bilaterally in a volume of 0.5 μl (0.25 μl per side) over 2.5 min at 30 min before anxiety-related behavioral tests. The injector tips were held in place for additional 5 min after the end of infusion to avoid backflow through the needle track.

For intra-VTA microinjection of AAV-DJ vectors containing the genes for Cre recombinase (AAV-Cre-GFP) or GFP alone (Vector Biolabs, Malvern, PA, USA), a total volume of 1.0 μl AAV vectors (0.5 μl per side) were injected bilaterally into the VTA (coordinates: 3.2 mm posterior to the bregma, 0.4 mm lateral to the midline, and 4.8 mm ventral to bregma) of adult AdipoR1^flox/flox^ mice after anesthetized, at a rate of 0.1 μl/min with a 33-gauge stainless-steel injector. Additional 5 min were allowed for diffusion and prevention of backflow. Anxiety-related behavioral tests or in vivo extracellular recording were conducted 21 days after AAV injection.

### Behavioral procedures

All behavioral tests were performed on male mice in the late light phase. Animals were transferred to the testing room and habituated for 3–4 h before beginning experiments. Mice were exposed to 2 h restraint stress and then received intra-VTA infusion of AdipoRon 1.5 h after the cessation of restraint stress. Behavioral tests assessing anxiety behavior were performed 30 min after intra-VTA injection. The animals were tested in both the elevated plus maze and the light–dark exploration test with an inter-test interval of at least 3 days to minimize potential carryover effects between behavioral tests. The experimenters who scored the behaviors were blind to animals’ genotypes and treatment conditions.

#### Acute restraint stress

Mice were transported to the procedure room in individual cages with some home cage bedding and habituated for 2 h without any disturbance. Restraint stress was performed in the animal's home cage by placing each mouse into a 50 ml polystyrene tube with ventilation for 30 min or 2 h. Control animals were briefly handled.

#### Elevated plus maze test

The elevated plus maze test is a validated and widely used anxiety paradigm in rodents, which is based on the natural conflict between the animal’s drive to explore a new environment and the tendency to avoid a potentially dangerous area [14, 15, 33–36]. The maze consisted of four arms arranged around  a central platform (5 × 5 cm^2^) that allowed access to all of the arms. Mice were placed in the central platform facing the corner between a closed arm and an open arm, and allowed to explore the elevated plus maze for 5 min. The test was recorded with a video camera mounted above the maze and connected to a computer. Behavioral performance was scored manually. The time spent on the open and closed arms and the numbers of entries made into each arm were measured. Entry was defined as all four paws entering one arm. The degree of anxiety was assessed by calculating the percentage of open arm time (time spent in the open arms/total time spent in all arms) and the percentage of open arm entries (entries into the open arms/total entries into all arms).

#### Light–dark box test

The light–dark test is based on a natural conflict of a mouse between the exploration of a novel environment and the innate aversion to brightly illuminated areas [14, 15, 34, 35]. The apparatus consists of a light (27 × 27 × 30 cm^3^) and a dark (18 × 27 × 30 cm^3^) compartment divided by a wall with a door between the two compartments. The light compartment was brightly illuminated with light intensity of 700 lux; and the dark compartment was black walled and covered at the top with black Plexiglas. Mice were placed individually in the center of the dark compartment facing away from the door. The latency for the mice to move to the light side and the time spent in the light compartment were recorded for 5 min with a video camera mounted above the light box. Entry to the light side was defined as all four paws entering the light compartment.

#### Locomotor activity

Locomotor activity was measured using the open field locomotor system (Omnitech Electronics Inc, Columbus, OH) [14, 15, 33–35]. The test apparatus consisted of an open field box (40 × 40 × 30 cm^3^) made of transparent acrylic surrounded by three sets of 16 photobeam arrays in the horizontal *x* and *y* axes as well as in the ventricle *z* axis. Locomotor movements were determined by breaks in photobeams and converted into distance traveled with the Fusion Software. Total distance traveled for 30 min was analyzed using the Fusion Software (Omnitech Electronics Inc, Columbus, OH).

### Electrophysiology

Mice were anesthetized with 4% chloral hydrate (400 mg/kg, intraperitoneally) and placed in a stereotaxic apparatus. Anesthesia was maintained by supplementary administration of chloral hydrate as required to suppress the limb compression withdrawal reflex. The core body temperature was sustained at 37 °C via a thermostatically controlled heating pad. Extracellular recording electrodes were pulled from borosilicate glass capillaries and filled with 2 M NaCl containing 2% Chicago sky blue dye (impedance 6–14 MΩ). The skull and dura were removed after an incision was made in the midline. Single-unit electrophysiological extracellular recording were performed as described previously [14]. Electrodes were lowered into the VTA (coordinates: 3.2 to 3.4 mm posterior to the bregma, 0.3 to 0.5 mm lateral to the midline, and 3.5 to 5.0 mm below the brain surface) using a micropositioner. Nine electrode tracks were made vertically throughout the VTA, each separated by 100 µm and arranged in a grid pattern. Spontaneously active dopamine neurons were identified with open filter settings (low cutoff = 30 Hz; high cutoff = 30 kHz) using established electrophysiological criteria [37, 38] and each neuron was recorded over a period of 3 min. Three parameters of dopamine neuron activity were measured for each mouse: (1) population activity, the number of spontaneously active dopamine neurons recorded per track; (2) average spontaneous firing rate (Hz); and (3) average percent burst firing, which is defined as the occurrence of two consecutive spikes with an interspike interval <80 ms, and the termination of a burst defined as two spikes with an interspike interval >160 ms [38]. The average firing rate and the average percent burst firing were obtained by averaging the values of firing rate and percent of burst firing for all dopamine neurons recorded in each individual mouse. At the end of the experiments, mice were decapitated and their brains processed for histological verification of electrode tracks.

For the experiments involving intra-VTA injection, vehicle (aCSF, 0.5 μl), adiponectin (0.15 μg, 0.5 μl), or AdipoRon (0.1 or 1.0 nmol, 0.5 μl) was infused into the VTA (coordinates: 3.3 mm posterior to the bregma, 0.4 mm lateral to the midline, and 4.2 mm ventral to bregma) with a 32 gauge Hamilton syringe at a rate of 0.5 μl/5 min. Five additional minutes were allowed for diffusion and prevention of backflow through the needle track before the injector was withdrawn. Six electrode tracks (3.2 to 3.4 mm posterior to the bregma; 0.3 to 0.5 mm lateral to the midline; 3.5–5.0 mm below the brain surface) were made vertically throughout the VTA.

### In situ hybridization

Radioactive in situ hybridization for detecting AdipoR1 and AdipoR2 gene expression were performed as previously described [29]. Briefly, brain sections were mounted on poly-lysine coated slides and sequentially fixed in 4% paraformaldehyde for 1 h, rinsed in 2 × SSC (300 mM NaCl, 30 mM Na citrate, pH 7.2), acetylated in 0.1 M triethanolamine (pH 8.0) with 0.25% (vol/vol) acetic anhydride for 10 min and dehydrated through a graded series of alcohol (50–100%). The tissue sections were then incubated with 70 μl of AdipoR1 or AdipoR2 cDNA probes labeled with S^35^-UTP and S^35^-CTP (PerkinElmer) at 55 °C overnight, rinsed in 2 × SSC and incubated in RNase A buffer (200 mg/ml) for 1 h at 37 °C followed by a series of washes with increasing stringency (2×, 1×, 0.5 × SSC). Finally, the sections were placed in 0.1 × SSC at 70 °C for 1 h, rinsed in distilled water, dehydrated in a graded series of alcohol, and exposed to X-ray film.

To examine whether AdipoR1 mRNA is colocalized with dopamine neurons in the VTA, we performed double in situ hybridization. cRNA probes complementary to either the mouse AdipoR1 mRNA or the mouse tyrosine hydroxylase (TH) mRNA were labeled with digoxigenin-11-UTP (for the AdipoR1 probe) or fluorescein-12-UTP (for the TH probe) using standard transcription methods. The brain sections were hybridized with a 1:1 mixture of the labeled probes at 55 °C overnight and subsequently rinsed in 2 × SSC, treated with RNase A (200 μg/ml) for 1 h at 37 °C, and washed in 2×, 1×, 0.5×, and 0.1 × SSC (for 5 min each), with a final rinse in 0.1 × SSC at 65 °C for 1 h. After the tissue was cooled to room temperature, it was processed for immunohistochemical staining for visualization of digoxigenin-11-labeled TH probe using the TSA Plus Fluorescence System kit (PerkinElmer). To accomplish this, brain sections were treated with 2% hydrogen peroxide in 0.05 M PBS for 30 min. The sections were then placed in a blocking solution (PerkinElmer) for 1 h followed by incubation with anti-fluorescence-HRP (Roche) overnight in a humidified chamber. The next day, the sections were rinsed in PBS and incubated with the fluorescein tyramide amplification reagent (PerkinElmer) for 15 min to reveal TH staining. For visualization of AdipoR1 mRNA, the sections were incubated with 2% hydrogen peroxide in 0.05 M PBS for 30 min, then rinsed in PBS followed by incubation with a sheep anti-digoxygenin antibody (Roche) in the blocking solution overnight. The sections were then rinsed and incubated for 1 h with anti-sheep-HRP secondary antibody in a humidified chamber. The sections were rinsed in PBS followed by incubation with the cyanine 3 tyramide amplification reagent (PerkinElmer) for 15 min. After rinsing, the slides were coverslipped immediately with the ProLong Gold antifade reagent (Invitrogen). For evaluation of the colocalization of AdipoR1 mRNA and TH mRNA, six consecutive sections through the VTA were analyzed. Signal specificity was ensured either by hybridization with sense-strand probes or pretreatment of brain sections with RNase A (200 μg/ml at 37 °C for 1 h).

### Immunofluorescence histochemistry

DAT-IRES-cre, Ai14 mice were perfused transcardially with 0.1 M phosphate-buffered saline (PBS) (pH 7.4) followed by 4% paraformaldehyde (PFA) in 0.1 M PBS. The brains  were removed and postfixed in 4% PFA at 4 °C for at least 24 h, and then transferred to 30% sucrose in PBS. Brains were sectioned to 40 μm coronal sections on a cryostat and stored in a cryoprotectant solution (30% sucrose, 30% ethylene glycol, 1% polyvinyl pyrolidone, 0.05 M sodium phosphate buffer). Brain sections were blocked for 1 h in a blocking buffer (1% BSA, 3% donkey serum and 0.3% Triton X-100 in PBS) and incubated with Rabbit anti-TH primary antibody (1:2000, #AB52, Millipore, Temecula, CA) overnight at 4 °C and then Alexa Fluor^®^ 488 donkey anti-Rabbit IgG antibody (1:400, A-21206, Invitrogen, Carlsbad, CA) for 1 h. Sections were mounted on coated slides and coverslipped using ProLong Gold antifade reagent. Expression of tdTomato and TH fluorescent staining and their colocolization were observed using Olympus fluorescence microscope.

### Western blot assay

Mice were decapitated rapidly, and trunk blood was collected in tubes containing 20 μl of 0.5% ethylenediaminetetraacetic acid disodium and centrifuged at 1000 × *g* for 10 min. The supernatant plasma was collected and stored at −20 °C. Western blotting for the measurement of adiponectin levels was performed as describe elsewhere [29, 33]. In brief, plasma samples were mixed with 5× sodium dodecyl sulfate–polyacrylamide gel electrophoresis loading dye (Beyotime Biotechnology, Shanghai, China) and denatured by boiling at 100 °C for 10 min. Denatured proteins (5 μl tenfold diluted plasma sample) were separated on an sodium dodecyl sulfate–polyacrylamide gel electrophoresis and transferred to polyvinylidene fluoride membrane. The membrane was blocked in Tris-buffered saline containing 1% dried milk and 0.1% Tween 20, and then incubated with anti-adiponectin antibody (Cat. #AF1119, 1:1000, R&D systems, Minneapolis, MN, USA) overnight at  4 °C. After washing, the membrane was incubated with a secondary antibody, donkey anti-goat IgG (Cat. #926-32214; 1:5000, LI-COR Biosciences, Lincoln, NE, USA). Signals were visualized and quantitatively analyzed.

### Histology

After the completion of behavioral tests, mice were killed by decapitation. Brains were sectioned at 40 μm and the placement of the cannula was verified. Animals with misplaced cannula were excluded from data analysis.

### Statistical analysis

The Shapiro–Wilk test was used to determine whether the data set was normally distributed. Statistical significance was assessed by two-tailed, unpaired or paired Student’s *t* test and one-way analysis of variance (ANOVA) for data with normal distribution and equal variance, or by the Mann–Whitney test, Wilcoxon signed-rank test, and Kruskal–Wallis test for data not following normal distribution or with unequal variance. Significant effects in the analysis of variances were followed up with Holm–Sidak (for parametric analysis) or Dunn’s (for non-parametric analysis) *post-hoc* tests. Results were considered significantly different when *P* < 0.05. All data were presented as mean ± standard error (s.e.m.).

## Results

### Expression of AdipoR1 in VTA dopamine neurons

**Fig. 1:**
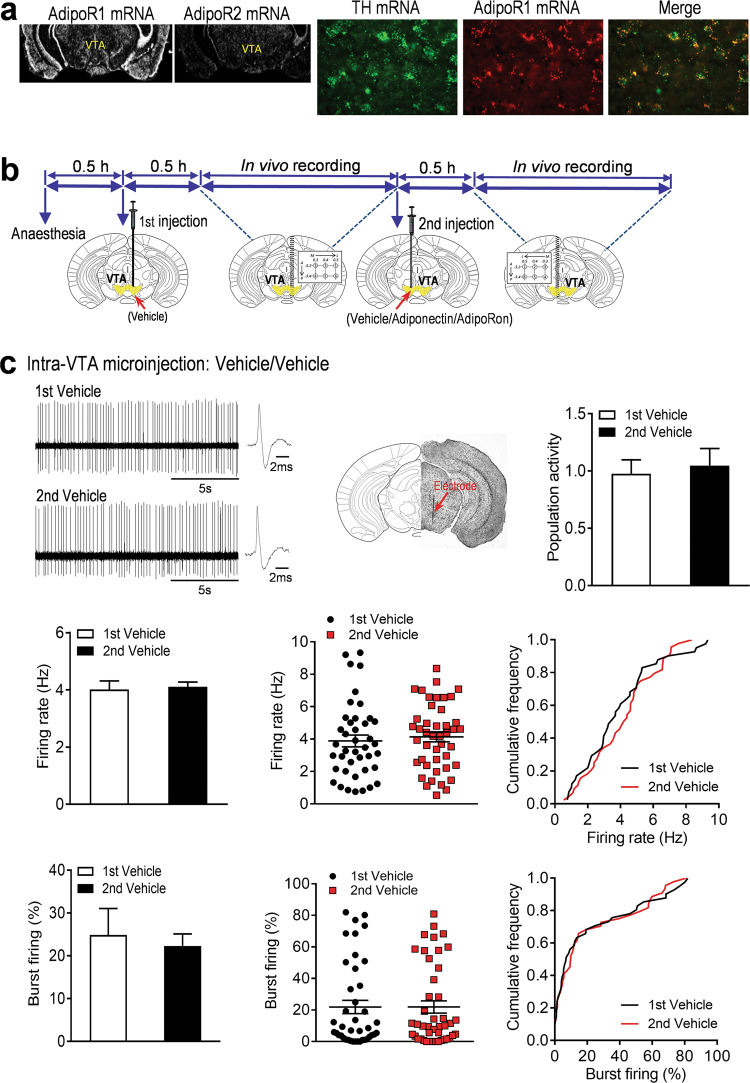

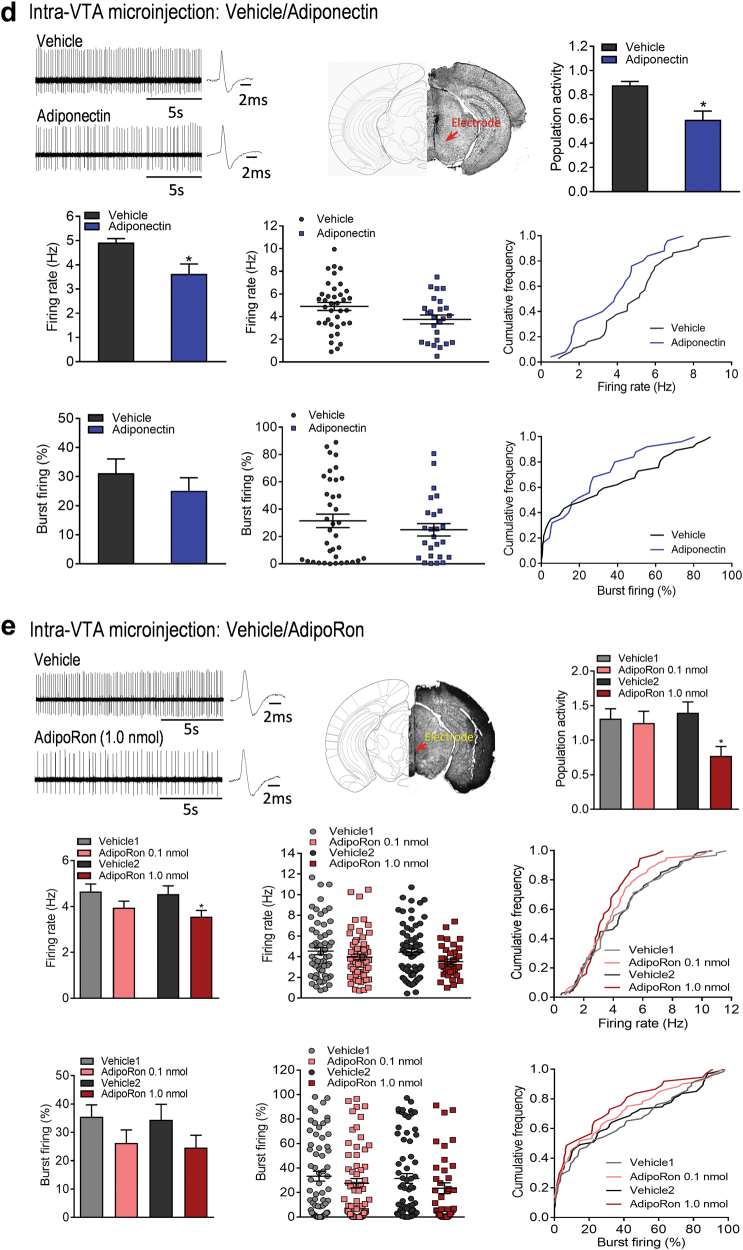
Effects of intra-VTA infusion of adiponectin and AdipoRon on dopamine neuron activity. (**a)** In situ hybridization showing expression patterns of AdipoR1 and AdipoR2 mRNA in the VTA (left) and colocalization of AdipoR1 and tyrosine hydroxylase (TH) mRNA (right). (**b)** Schematic procedure of in vivo extracellular recording of dopamine neurons in the VTA. (**c)** Effects of intra-VTA vehicle injection. Top-left, representative extracellular voltage traces from identified dopamine neurons in the VTA; top-center, representative image demonstrating the electrode track through the VTA; top-right, number of spontaneously active dopamine neurons per track in the VTA. Middle-left, firing rate; middle-center, scatter plot distribution; middle-right, cumulative frequency distribution. Bottom-left, percent burst firing; bottom-center, scatter plot distribution; bottom-right, cumulative frequency distribution. 1st vehicle: *n* = 7 mice; 2nd vehicle: *n* = 7 mice. (**d)** Effects of intra-VTA adiponectin injection. Top-left, representative extracellular voltage traces from VTA dopamine neurons; top-center, representative image demonstrating the electrode track through the VTA; top-right, number of spontaneously active dopamine neurons per track in the VTA. Middle-left, firing rate; middle-center, scatter plot distribution; middle-right, cumulative frequency distribution. Bottom-left, percent burst firing; bottom-center, scatter plot distribution; bottom-right, cumulative frequency distribution. *P < 0.05, **P < 0.01 compare with the vehicle-treated control group. Vehicle: *n* = 7 mice; adiponectin: *n* = 7 mice. (**e)** Effects of intra-VTA AdipoRon injection. Top-left, representative extracellular voltage traces from VTA dopamine neurons; top-center, representative image demonstrating the electrode track through the VTA; top-right, number of spontaneously active dopamine neurons per track in the VTA. Middle-left, firing rate; middle-center, scatter plot distribution; middle-right, cumulative frequency distribution. Bottom-left, percent burst firing; bottom-center, scatter plot distribution; bottom-right, cumulative frequency distribution. *P < 0.05, **P < 0.01 compare with the vehicle-treated group. Vehicle 1: *n* = 8 mice; AdipoRon 0.1 nmol: *n* = 8 mice; Vehicle 2: *n* = 8 mice; AdipoRon 1.0 nmol: *n* = 8 mice. VTA ventral tegmental area

To determine whether AdipoR1 and AdipoR2 are expressed in VTA dopamine neurons, we first performed in situ hybridization with radioactive cRNA probes to examine the expression patterns of AdipoR1 and AdipoR2 mRNA in the VTA. We found that AdipoR1 mRNA was abundant in the VTA, whereas AdipoR2 mRNA was almost undetectable in this region (Fig. 1a). Furthermore, double-labeling in situ hybridization with non-radioactive fluorescein-labeled and digoxigenin-labeled cRNA probes revealed that the vast majority of dopamine neurons labeled with TH in the VTA expressed AdipoR1 mRNA (Fig. 1a). This finding provides the anatomical basis for a possible modulation of dopamine neuron activity by adiponectin/AdipoR1 signaling.

### Inhibition of VTA dopamine neuron firing by local infusion of adiponectin and AdipoRon

Given the expression of AdipoR1 in VTA dopamine neurons, we tested whether local intra-VTA infusion of full-length adiponectin modulates the firing properties of dopamine neurons. The spontaneous firing activity of putative VTA dopamine neurons in anesthetized wild-type mice was recorded using the in vivo extracellular single-unit recording technique. In order to reduce error variance  due to individual differences, we used a within-subjects design, in which dopamine neurons in the VTA were recorded after two separate, sequential infusions in the two sides of the VTA of each mouse (Fig. 1b). First, we tested whether the temporal sequence of two intra-VTA infusion recordings could be a potential confounding variable. We compared spontaneous dopamine neuron firing activity recorded following the first vs. second intra-VTA vehicle (aCSF) infusion. As shown in Figure 1c, there were no significant differences in dopamine neuron population activity, the average firing rate and average percentage of burst firing between the first and second intra-VTA vehicle infusion (0.5 μl/side; number of spontaneously active dopamine neurons per electrode track: 1st Vehicle, 0.98 ± 0.12 neurons/track, total 41 neurons from seven mice; 2nd Vehicle, 1.05 ± 0.15 neurons/track, total 44 neurons from seven mice; *t*_(6)_ = 0.609, *P* = 0.565; average firing rate: 1st Vehicle, 4.02 ± 0.30 Hz; 2nd Vehicle, 4.11 ± 0.16 Hz; *t*_(6)_ = 0.321, *P* = 0.759; percentage of burst firing: 1st Vehicle, 24.84 ± 6.22%, 37 neurons; 2nd Vehicle, 22.32 ± 2.82%; 39 neurons; Wilcoxon signed-rank test, *P* = 0.938).

Following intra-VTA infusion of adiponectin (0.15 μg in 0.5 μl), the number of spontaneously active dopamine neurons per electrode track was significantly decreased in comparison with the vehicle aCSF) treated group (Vehicle: 0.88 ± 0.03 neurons/track, total 37 neurons from seven mice; Adiponectin: 0.60 ± 0.07 neurons/track, total 25 neurons from seven mice; Wilcoxon signed-rank test, *P* = 0.031; Fig. 1d). The average firing rate of VTA dopamine neurons was also reduced by intra-VTA infusion of adiponectin (Vehicle: 4.92 ± 0.16 Hz; Adiponectin: 3.63 ± 0.40 Hz; *t*_(6) = _2.865, P = 0.029; Fig. 1d). However, and the average percentage of burst firing was not significantly different between adiponectin and vehicle injections (Vehicle: 31.20 ± 4.88%, 36 neurons; Adiponectin: 25.13 ± 4.47%, 25 neurons; *t*_(6) = _0.992, *P* = 0.359; Fig. 1d). These results suggest that adiponectin in the VTA inhibits dopamine neuron firing activity.

To further confirm these findings, we examined the effects of AdipoRon, a small-molecule adiponectin mimetic that binds both AdipoR1 and AdipoR2 with high affinity, on dopamine neuron firing patterns [39]. Similar to the observations with adiponectin, intra-VTA infusion of AdipoRon reduced the number of spontaneously active dopamine neurons per track (Vehicle1: 1.31 ± 0.14 neurons/track, total 63 neurons from eight mice; AdipoRon 0.1 nmol: 1.25 ± 0.17 neurons/track, total 60 neurons from eight mice; *t*_(7)_ = 0.263, *P* = 0.800. Vehicle2: 1.40 ± 0.16 neurons/track, total 67 neurons from eight mice; AdipoRon 1.0 nmol: 0.77 ± 0.14 neurons/track, total 37 neurons from eight mice; Wilcoxon signed-rank test, *P* = 0.023) and average firing rate (Vehicle1: 4.65 ± 0.33 Hz; AdipoRon 0.1 nmol: 3.95 ± 0.28 Hz; *t*_(7)_ = 2.129, *P* = 0.071. Vehicle2: 4.54 ± 0.36 Hz; AdipoRon 1.0 nmol: 3.56 ± 0.27 Hz; *t*_(7)_ = 3.362, *P* = 0.012) in a dose-dependent manner (Fig. 1e). The percentage of burst firing was not significantly altered by AdipoRon (Vehicle1: 35.5 ± 4.22%, 57 neurons; AdipoRon 0.1 nmol: 26.26 ± 4.60%,  52 neurons; *t*_(7)_ = 1.652, *P* = 0.143. Vehicle2: 34.4 ± 5.46%, 63 neurons; AdipoRon 1.0 nmol: 24.62 ± 4.36%, 33 neurons; *t*_(7)_ = 1.373, *P* = 0.212. Figure 1e). These results suggest that activation of AdipoR, possibly the AdipoR1 subtype, in the VTA exerts inhibitory effects on dopamine neuron firing.

### Intra-VTA infusion of AdipoRon reverses acute restraint stress-induced increase in VTA dopamine neuron firing

**Fig. 2:**
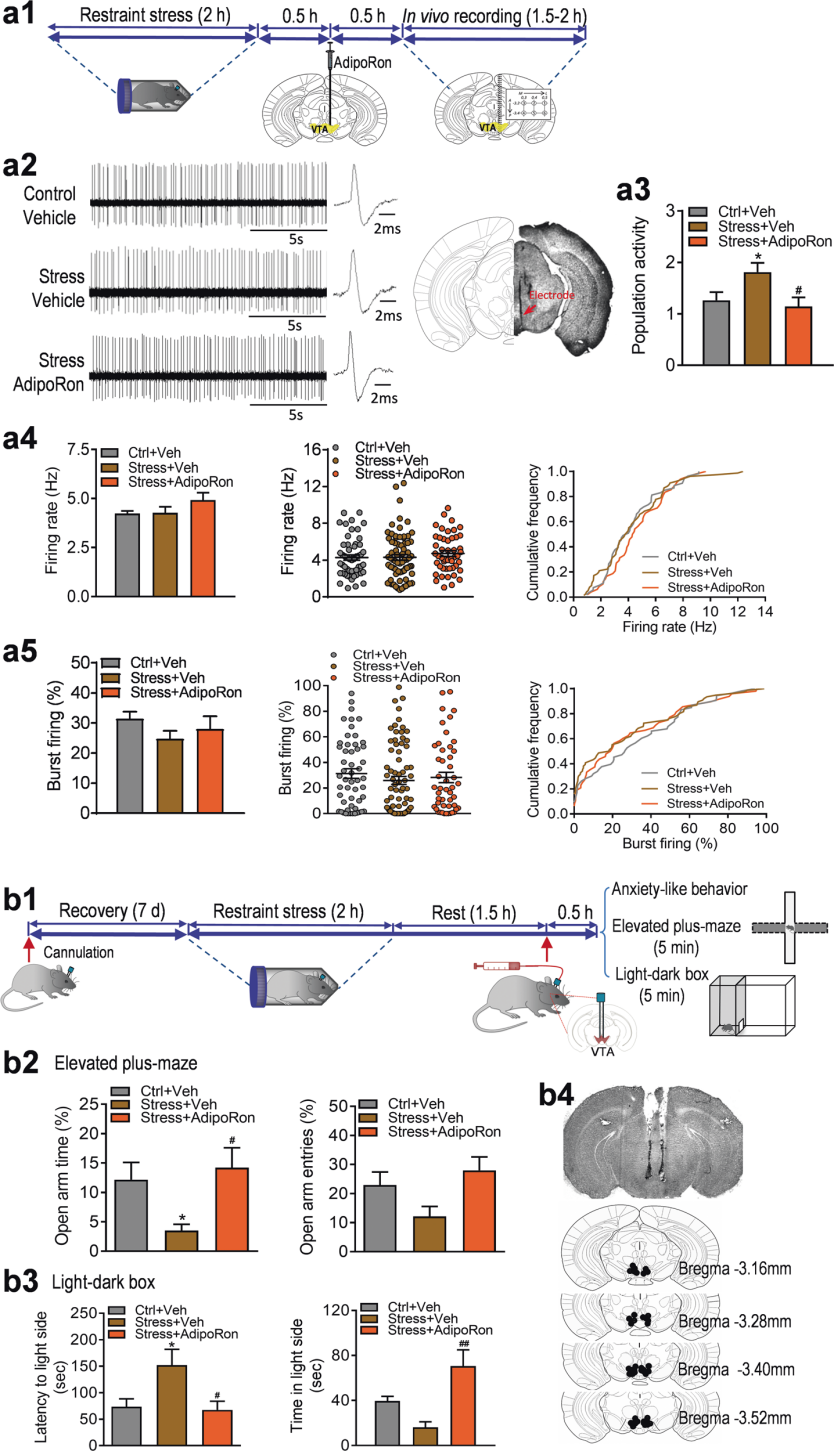
Effect of AdipoRon on acute restraint stress-induced effects on VTA dopamine neuron activity and anxiogenic-like response. (**a1**) Schematic timeline of acute restraint stress, intra-VTA microinjection and in vivo extracellular recordings. (**a2**) Left, representative extracellular voltage traces from dopamine neurons in the VTA; right, representative image demonstrating the electrode track through the VTA. (**a3**) Number of spontaneously active dopamine neurons per track through the VTA. (**a4**) Firing rate: bar graph (left), scatter plot (middle) and cumulative frequency distribution (right). (**a5**) Proportion of burst firing: bar graph (left), scatter plot (middle) and cumulative frequency distribution (right). Control + vehicle: *n* = 7 mice, 53 neurons; restraint stress + vehicle: *n* = 7 mice, 76 neurons; restraint stress + AdipoRon: *n* = 7 mice, 48 neurons. (**b1**) Schematic timeline of acute restraint and behavioral testing procedures. (**b2**) Elevated plus maze test. Left, percentage of open/total arm time. Right, percentage of open/total arm entries. Control + vehicle: *n* = 10 mice; restraint stress + vehicle: *n* = 10 mice; restraint stress + AdipoRon: *n* = 10 mice. (**b3**) Light–dark box test. Left, latency to enter the light side. Right, time spent in the light compartment. Control + vehicle: *n* = 8 mice; restraint stress + vehicle: *n* = 6 mice; restraint stress + AdipoRon: *n* = 8 mice. (**b4**) Representative image (upper) and schematic illustration of injection sites in the VTA (lower). *P < 0.05 compared with the non-stressed, vehicle-treated group. ^#^*P* < 0.05, ^##^*P* < 0.01 compared with the stressed, vehicle-treated group

Previous reports have demonstrated that VTA dopamine neurons are excited during acute restraint stress in rats [5, 7]. We examined whether a similar effect can be induced by restraint stress in mice and whether such effect can be reversed by AdipoRon. Wild-type mice were exposed to 2 h of restraint stress or brief handling followed by intra-VTA infusion of AdipoRon prior to in vivo extracellular recordings of VTA dopamine neurons (Fig. 2a1). We found that restraint stress increased the number of spontaneously active dopamine neurons in the VTA, and this effect was significantly reversed by intra-VTA infusion of AdipoRon (control + vehicle: 1.26 ± 0.16 neurons/track, total 53 neurons from seven mice; restraint stress + vehicle: 1.81 ± 0.18 neurons/track, total 76 neurons from seven mice; restraint stress + AdipoRon: 1.14 ± 0.18 neurons/track, total 48 neurons from seven mice; one-way ANOVA, F_(2,18)_ = 4.295, *P* = 0.030; Fig. 2a3). There were no significant differences in average firing rate (control + vehicle: 4.23 ± 0.13 Hz; restraint stress + vehicle: 4.27 ± 0.31 Hz; restraint stress + AdipoRon: 4.92 ± 0.37 Hz; one-way ANOVA, F_(2,18)_ = 1.789, *P* = 0.196; Fig. 2a4) and percentage of burst firing (control + vehicle: 31.40 ± 2.34%, 48 neurons; restraint stress + vehicle: 24.71 ± 2.65%, 64 neurons; restraint stress + AdipoRon: 27.99 ± 4.26%, 45 neurons; one-way ANOVA, F_(2,18)_ = 1.099, *P* = 0.355; Fig. 2a5) between two treatment groups.

### Adiponectin reverses acute restraint stress-induced anxiogenic responses

Changes in VTA dopamine neuron activity have been associated with stress-induced anxiety [2, 7, 40, 41]. We first asked whether central administration of adiponectin affects anxiety behavior. Wild-type mice received intracerebroventricular (ICV) injection of adiponectin (0.1, 0.3 µg) 30 min before the elevated plus maze and light/dark box tests to assess anxiety-like behavior. We found that adiponectin treatment significantly increased the percentage of open/total arm entries (F_(2,39)_ = 4.081, *P* = 0.025) and induced a trend toward an increase in the percentage of open/total arm time (Kruskal–Wallis test, *P* = 0.119) in the elevated plus maze test (Supplementary Fig. 1a). Also, an increase in the time spent in the light compartment during the light/dark box test was observed after ICV infusion of adiponectin (F_(2,21)_ = 4.857, *P* = 0.019; Supplementary Fig. 1b). These data suggest a central anxiolytic action of adiponectin. We next examined whether restraint stress-induced anxiogenic behavior can be attenuated by intra-VTA infusion of AdipoRon. Mice were first exposed to 2 h of restraint stress, then AdipoRon was infused into the VTA bilaterally 30 min before testing (Fig. 2b1). The elevated plus maze and light/dark box tests were performed at 2 h after cessation of restraint stress. Restraint stress significantly reduced the percentage of open/total arm time, whereas intra-VTA injection of AdipoRon in stressed mice increased the percentage of both open/total arm time and entries in the elevated plus maze test (open/total arm time: F_(2,27)_ = 4.534, *P* = 0.020; open/total arm entries: Kruskal–Wallis test, *P* = 0.073; Fig. 2b2). In the light/dark box test, restraint stress increased the latency to the light side and decreased the time spent in the light compartment; these effects were reversed by intra-VTA infusion of AdipoRon (latency to the light side: Kruskal–Wallis test, *P* = 0.014; time in the light side: F_(2, 19)_ = 7.531, *P* = 0.004; Fig. 2b3).These results indicate that activation of adiponectin receptors in the VTA is sufficient to reverse stress-induced anxiety, in parallel with its inhibition of spontaneous firing activity of dopamine neurons.

### Adiponectin insufficiency increases VTA dopamine neuron firing activity and induces anxiogenic behavior

**Fig. 3:**
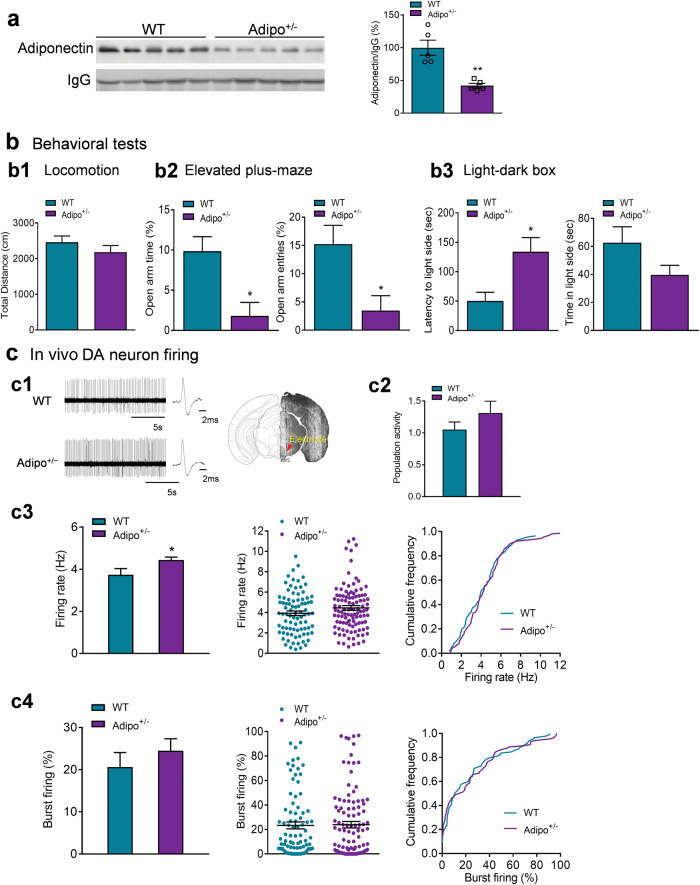
Effects of adiponectin insufficiency on anxiety-like behavior and VTA dopamine neuron activity. (**a)** Plasma adiponectin levels. *n* = 5 mice per group. (**b)** Behavioral tests. (**b1)** Locomotor activity. WT: *n* = 11 mice; Adipo^+/-^: *n* = 10 mice. (**b2)** Elevated plus maze test. Left, percentage of open/total arm time. Right, percentage of open/total arm entries. WT: *n* = 11 mice; Adipo^+/-^: *n* = 9 mice. (**b3)** Light–dark box test. Left, latency to enter the light side; right, time spent in the light compartment. WT: *n* = 11 mice; Adipo^+/-^: *n* = 21 mice. (**c1)** Left, representative extracellular voltage traces from VTA dopamine neurons; right, representative image demonstrating the electrode track through the VTA. (**c2)** Number of spontaneously active dopamine neurons per track. (**c3)** Firing rate: bar graph (left), scatter plot (middle) and cumulative frequency distribution (right). (**c4)** Percentage of burst firing: bar graph (left), scatter plot (middle) and cumulative frequency distribution (right). WT: *n* = 9 mice; Adipo^+/-^: *n* = 9 mice. *P *<* 0.05, **P < 0.01 compared with wild-type littermate control (WT) mice

Next, we investigated whether reduced endogenous adiponectin levels in mice affect VTA dopamine neuron firing activity and anxiety-related behavior. As shown previously [29, 32, 42], adiponectin-haploinsufficient (Adipo^+/-^) mice have 42% of normal plasma adiponectin levels (wild-type: 100 ± 11.62%, *n* = 5 mice; Adipo^+/-^: 42.00 ± 3.48%, *n* = 5 mice; *t*_(8)_ = 4.780, *P* = 0.001; Fig. 3a), representing a model with endogenous adiponectin insufficiency. Adipo^+/-^ mice showed normal locomotor activity in the open field test (*t*_(19)_ = 1.079, *P* = 0.294; Fig. 3b1), decreased percentage of open/total arm time and entries in the elevated plus maze test (open/total arm time: Mann–Whitney test, *P* = 0.015; open/total arm entries: Mann–Whitney test, *P* = 0.015; Fig. 3b2) and increased latency to the light side in the light/dark box test (latency to the light side: Mann–Whitney test, *P* = 0.027; time in the light side: Mann–Whitney test, *P* = 0.130; Fig. 3b3), indicating an anxiogenic phenotype.

To determine if increased anxiety in Adipo^+/-^ mice is correlated with changes in VTA dopamine neuron firing activity, we performed in vivo extracellular recordings of VTA dopamine neurons from Adipo^+/-^ mice and their wild-type littermate controls. Adipo^+/-^ mice exhibited normal population activity of dopamine neurons (WT: 1.05 ± 0.12 neurons/track, total 85 neurons from nine mice; Adipo^+/-^: 1.31 ± 0.19 neurons/track, total 106 neurons from nine mice; *t*_(16) = _1.171, *P* = 0.259; Fig. 3c2) and percentage of burst firing (WT: 20.63 ± 3.43%, 78 neurons; Adipo^+/-^: 24.52 ± 2.83%, 93 neurons; *t*_(16) = _0.875, *P* = 0.394; Fig. 3c4), but a significant increase in the firing rate (WT: 3.73 ± 0.29 Hz; Adipo^+/-^: 4.44 ± 0.13 Hz; *t*_(16) = _2.181, *P* = 0.044; Fig. 3c3).

### Deletion of AdipoR1 in the VTA increases anxiety-like behavior

To determine if AdipoR1 is involved in mediating anxiety-like behavior, AdipoR1^flox/flox^ mice received bilateral infusion of AAV-Cre-GFP or AAV-GFP into the VTA and were subjected to the elevated plus maze and light/dark box tests 3 weeks after AAV injection. Mice injected with AAV-Cre-GFP in the VTA displayed a decreased tendency in the percentage of open arm time (Mann–Whitney test, *P* = 0.072) and the percentage of open arm entries (*t*_(12)_ = 1.943, *P* = 0.076) (Supplementary Fig. 2). In the light–dark box test, mice injected with AAV-Cre-GFP explored the light compartment to a lesser extent than mice injected with AAV-GFP (latency to enter the light side: Mann–Whitney test, *P* = 0.336; time spent in the light side: *t*_(13)_ = 2.169, *P* = 0.049) (Supplementary Fig. 2). These observations suggest that AdipoR1 in the VTA may play an important role in mediating anxiety-related behaviors.

### Deletion of AdipoR1 specifically in dopamine neurons results in anxiogenic behavior

**Fig. 4:**
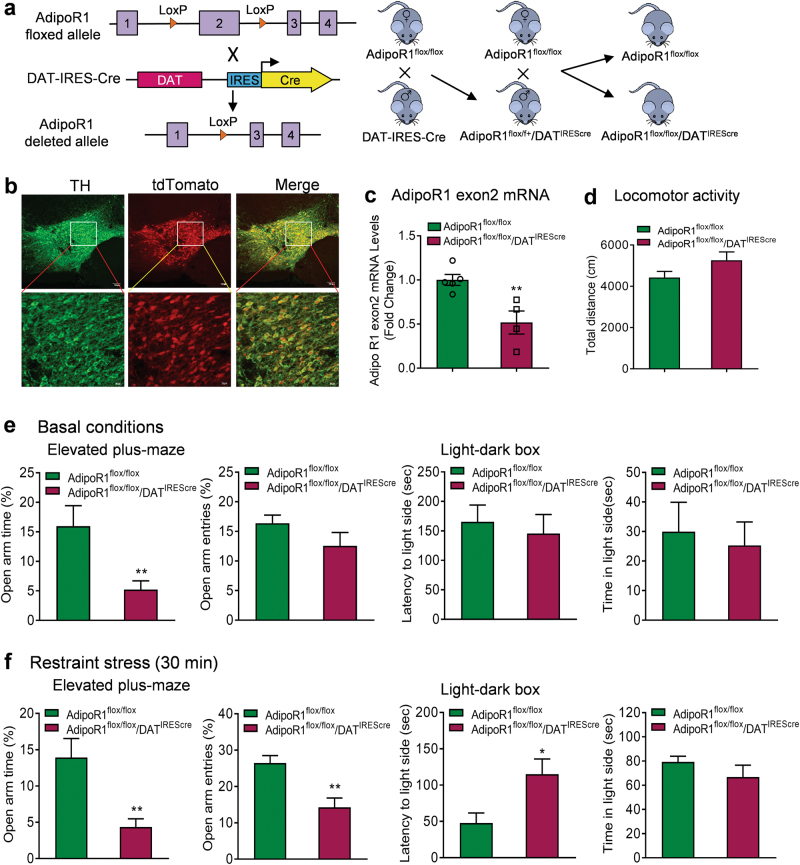

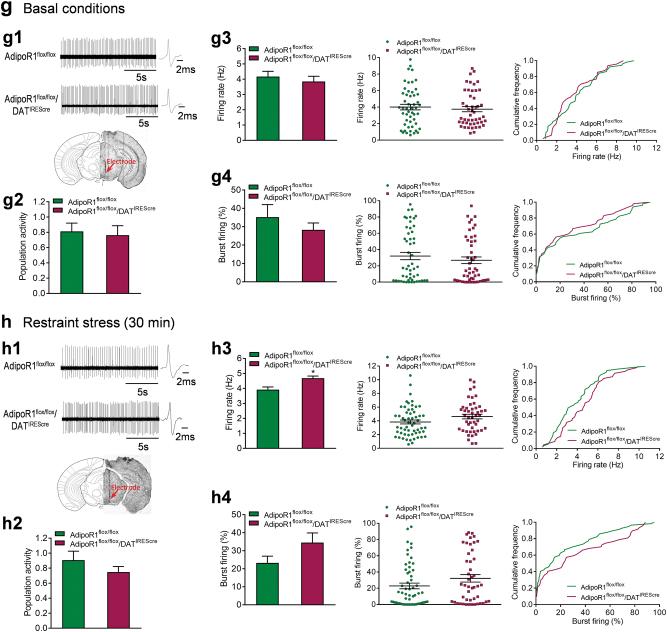
Effects of AdipoR1 deletion in dopamine neurons on anxiety-like behavior and VTA dopamine neuron activity. (**a)** Schematic diagram of generation of mice with AdipoR1 deletion in dopamine neurons. (**b)** Colocalization of DAT-IRES-cre (tdTomato fluorescence, red) and tyrosine hydroxylase (TH, green). (**c**) Real-time PCR analysis showing the Cre-mediated deletion of exon 2 of AdipoR1 in the VTA of AdipoR1^flox/flox^/DAT^IREScre^ mice. AdipoR1^flox/flox^: *n* = 5 mice; AdipoR1^flox/flox^/DAT^IREScre^: *n* = 4 mice. (**d)** Locomotor activity. AdipoR1^flox/flox^: *n* = 14 mice; AdipoR1^flox/flox^/DAT^IREScre^: *n* = 12 mice. (**e)** Behavioral tests for anxiety under basal non-stress conditions. Left and middle-left, elevated plus maze test. AdipoR1^flox/flox^: *n* = 14 mice; AdipoR1^flox/flox^/DAT^IREScre^: *n* = 11 mice. Middle-right and right, light–dark box test. AdipoR1^flox/flox^: *n* = 14 mice; AdipoR1^flox/flox^/DAT^IREScre^: *n* = 12 mice. (**f)** Behavioral tests for anxiety after 30-min restraint stress. Left and middle-left, elevated plus maze test. AdipoR1^flox/flox^: *n* = 9 mice; AdipoR1^flox/flox^/DAT^IREScre^: *n* = 7 mice. Middle-right and right, light–dark box test. AdipoR1^flox/flox^: *n* = 8 mice; AdipoR1^flox/flox^/DAT^IREScre^: *n* = 7 mice. (**g)** Firing activities of VTA dopamine neurons under basal non-stress conditions. (**g1**) Upper, representative extracellular voltage traces from dopamine neurons in the VTA; lower, representative image demonstrating the electrode track through the VTA. (**g2**) Number of spontaneously active dopamine neurons per track. (**g3**) Left, firing rate; middle, scatter plot distribution; right, cumulative frequency distribution. (**g4**) Left, proportion of burst firing; middle, scatter plot distribution; right, cumulative frequency distribution. AdipoR1^flox/flox^: *n* = 7 mice, AdipoR1^flox/flox^/DAT^IREScre^: *n* = 7 mice. (**h)** Firing activities of VTA dopamine neurons after 30-min restraint stress. (**h1**) Upper, representative extracellular voltage traces from dopamine neurons in the VTA; lower, representative image demonstrating the electrode track through the VTA. (**h2**) Number of spontaneously active dopamine neurons per track. (**h3**) Left, firing rate; middle, scatter plot distribution; right, cumulative frequency distribution. (**h4**) Left, proportion of burst firing; middle, scatter plot distribution; right, cumulative frequency distribution. AdipoR1^flox/flox^: *n* = 7 mice, AdipoR1^flox/flox^/DAT^IREScre^: *n* = 7 mice. *P *<* 0.05, **P < 0.01 compared with AdipoR1^flox/flox^ littermate controls

To determine the physiological relevance of adiponectin/AdipoR1 signaling in dopamine neurons to anxiety-related behavior, we generated mice lacking AdipoR1 specifically in dopamine neurons using the Cre–loxP system. The DAT-IRES-cre mice have Cre recombinase expression directed to dopaminergic neurons, without disrupting endogenous dopamine transporter expression [43]. The specificity of DAT-IRES-cre-mediated recombination was confirmed by crossing DAT-IRES-cre mice with a tdTomato reporter line (Ai14). tdTomato fluorescence was exclusively colocalized with the dopamine neuron marker TH (Fig. 4b). To delete AdipoR1 from dopamine neurons, DAT-IRES-cre mice were crossed with AdipoR1^flox/flox^ mice that possess loxP sites flanking exon 2 of the *AdipoR1* gene [31]. The deletion of exon 2 causes a frameshift that results in null mutation of AdipoR1 [31]. Cre-mediated excision of AdipoR1 exon 2 in the VTA of AdipoR1^flox/flox^/DAT^IREScre^ mice was confirmed by real-time PCR showing reduced exon 2 mRNA levels (*t*_(7)_ = 3.585, *P* = 0.009; Fig. 4c). The microdissections of the VTA unavoidably contained non-dopamine neurons and adjacent tissue, which may account for the residual exon 2 expression.

AdipoR1^flox/flox^/DAT^IREScre^ mice showed normal locomotor activity in the open field test compared with littermate AdipoR1^flox/flox^ controls (*t*_(24)_ = 1.710, *P* = 0.100; Fig. 4d). Under basal (non-stress) conditions, AdipoR1^flox/flox^/DAT^IREScre^ mice displayed a decrease in the percentage of open/total arm time (Mann–Whitney test, *P* = 0.009), but no significant change in the percentage of open/total arm entries (Mann–Whitney test, *P* = 0.442) in the elevated plus maze test (Fig. 4e). In the light/dark box test, AdipoR1^flox/flox^/DAT^IREScre^ mice showed normal latency to the light side (*t*_(24)_ = 0.465, *P* = 0.646) and the time spent in the light compartment (Mann–Whitney test, *P* = 0.733) (Fig. 4e). We further determined if deletion of AdipoR1 from dopamine neurons increases the sensitivity to stress. AdipoR1^flox/flox^/DAT^IREScre^ mice and control mice were subjected to short-term (30 min) restraint stress before behavioral testing. AdipoR1^flox/flox^/DAT^IREScre^ mice exhibited enhanced anxiety-like behavior in response to restraint stress as indicated by reduced percentages of open/total arm time and entries in the elevated plus maze test (open/total arm time: Mann–Whitney test, *P* = 0.005; open/total arm entries: *t*_(13)_ = 3.738, *P* = 0.003) and increased latency to the light side in the light/dark box test (latency to the light side: *t*_(12)_ = 2.787, *P* = 0.016; time in the light side: *t*_(12)_ = 1.262, *P* = 0.231) compared to AdipoR1^flox/flox^ control mice (Fig. 4f). These data demonstrate that AdipoR1 signaling in dopamine neurons is critical for the modulation of anxiety-like behavior in response to stress.

To determine whether the insertion of loxP sites affects anxiety behavior, AdipoR1^flox/flox^ mice and wild-type mice were subjected to the elevated plus maze and light/dark box tests. No differences were observed in these two tests between AdipoR1^flox/flox^ mice and wild-type mice (Elevated plus maze: open/total arm time: *t*_(11)_ = 0.180, *P* = 0.860; open/total arm entries: *t*_(11)_ = 0.810, *P* = 0.435; Light/dark box: latency to enter the light side: *t*_(13)_ = 0.526, *P* = 0.608; time spent in the light side: *t*_(13)_ = 0.490, *P* = 0.632) (Supplementary Fig. 3).

### Targeted deletion of AdipoR1 in dopamine neurons potentiates VTA dopamine neuron response to stress

To understand the role of adiponectin/AdipoR1 signaling in modulating VTA dopamine neuronal activity, we examined the firing properties of VTA dopamine neurons in AdipoR1^flox/flox^/DAT^IREScre^ mice and AdipoR1^flox/flox^ littermate controls. In vivo recordings within the VTA revealed no difference in the number of spontaneously active dopamine neurons, firing rate and percentage of burst firing between the two genotypes (number of active neurons: AdipoR1^flox/flox^: 0.81 ± 0.11 neurons/track; AdipoR1^flox/flox^/DAT^IREScre^: 0.76 ± 0.12 neurons/track; Mann–Whitney test, *P* = 0.679; firing rate: AdipoR1^flox/flox^: 4.17 ± 0.35 Hz; AdipoR1^flox/flox^/DAT^IREScre^: 3.85 ± 0.34 Hz; *t*_(12)_ = 0.655, *P* = 0.525; percent burst firing: AdipoR1^flox/flox^: 35.22 ± 6.84%, 47 neurons; AdipoR1^flox/flox^/DAT^IREScre^: 28.30 ± 3.76%, 43 neurons; Mann–Whitney test, *P* = 0.456; AdipoR1^flox/flox^, 51 neurons from seven mice; AdipoR1^flox/flox^/DAT^IREScre^ 48 neurons from seven mice; Fig. 4g). Following exposure to 30 min of restraint stress, AdipoR1^flox/flox^/DAT^IREScre^ mice exhibited significantly higher firing rate of VTA dopamine neurons compared to AdipoR1^flox/flox^ littermate controls (AdipoR1^flox/flox^: 3.92 ± 0.18 Hz; *n* = 7 mice, 57 neurons; AdipoR1^flox/flox^/DAT^IREScre^: 4.68 ± 0.14 Hz; *n* = 7 mice, 47 neurons; Mann–Whitney test, *P* = 0.018; Fig. 4h3) although spontaneously active DA neuron population and percentage of burst firing remained no difference (population activity: AdipoR1^flox/flox^: 0.91 ± 0.12 neurons/track; AdipoR1^flox/flox^/DAT^IREScre^: 0.75 ± 0.08 neurons/track; *t*_(12)_ = 1.110, *P* = 0.289; percent burst firing: AdipoR1^flox/flox^: 23.20 ± 3.78% (50 neurons); AdipoR1^flox/flox^/DAT^IREScre^: 34.49 ± 5.36% (43 neurons); *t*_(12)_ = 1.722, *P* = 0.111; Fig. 4h2 and Fig. 4h4). These changes in dopamine neuron firing activity are in line with the anxiety-like behavioral phenotype of AdipoR1^flox/flox^/DAT^IREScre^ mice.

### AdipoR1 mediates adiponectin action on VTA dopamine neuron activity

**Fig. 5:**
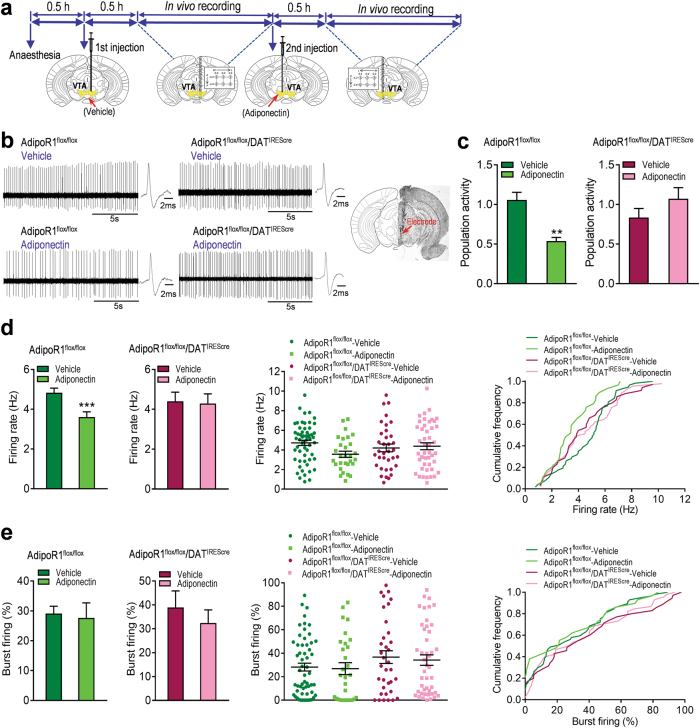

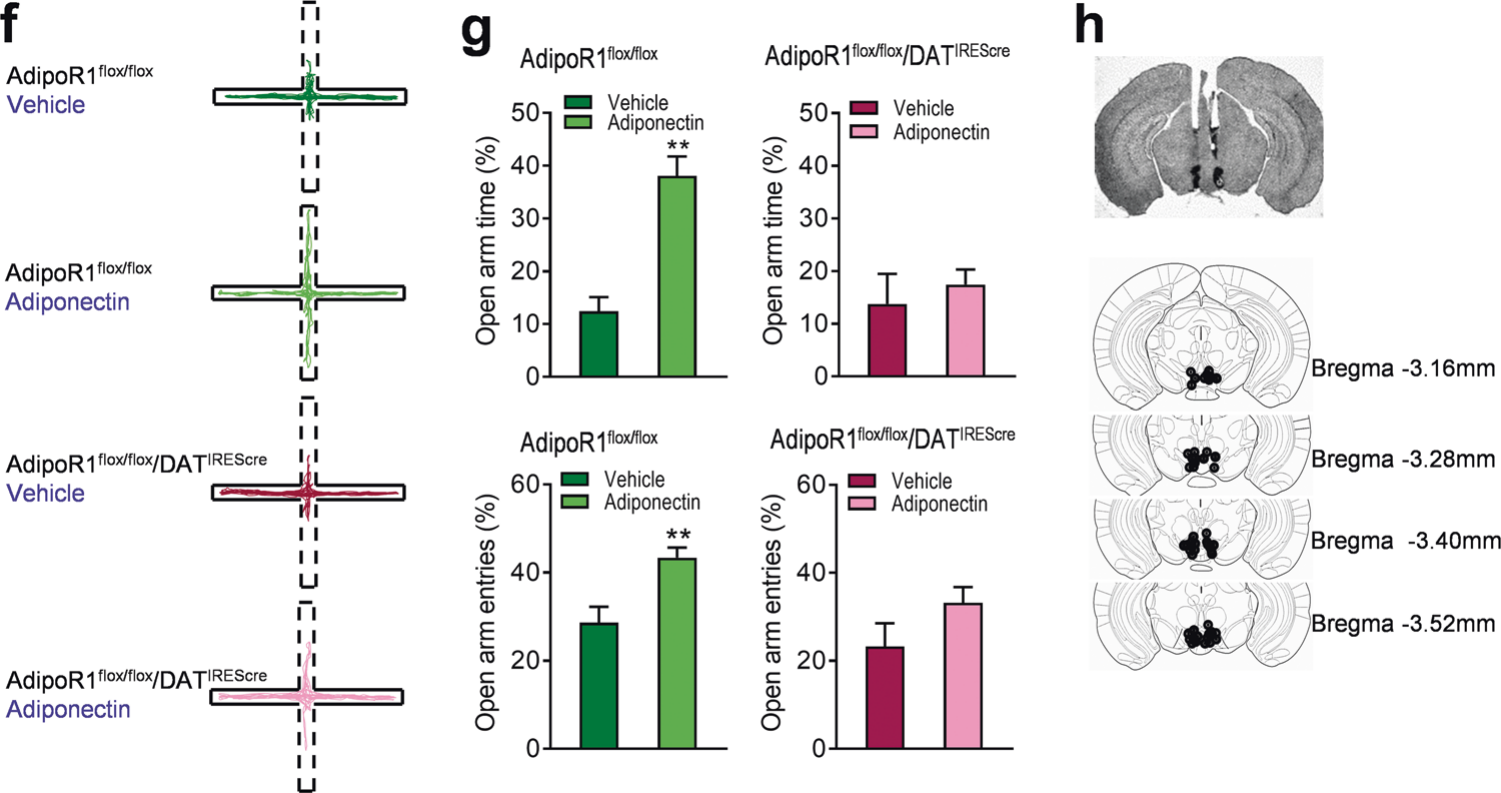
AdipoR1 mediates the effects of adiponectin on VTA dopamine neuron activity and anxiety-like behavior. (**a)** Schematic procedure of in vivo extracellular recording of dopamine neurons in the VTA. (**b)** Left, representative extracellular voltage traces from VTA dopamine neurons; right, representative image demonstrating the electrode track through the VTA. (**c)** Number of spontaneously active dopamine neurons per track in the VTA. (**d)** Left and middle-left, firing rate; middle-right, scatter plot distribution; right, cumulative frequency distribution. (**e)** Left and middle-left, percentage of burst firing; middle-right, scatter plot distribution; right, cumulative frequency distribution. AdipoR1^flox/flox^ + vehicle: *n* = 9 mice, AdipoR1^flox/flox^ + adiponectin: *n* = 9 mice, AdipoR1^flox/flox^/DAT^IREScre^ + vehicle: *n* = 7 mice; AdipoR1^flox/flox^/DAT^IREScre^ + Adiponectin: *n* = 7 mice. (**f)** Representative traces of the elevated plus maze test in four different treatment groups. (**g)** Top, percentage of open/total arm time; bottom, percentage of open/total arm entries. (**h)** Top, representative picture showing injection sites; bottom, schematic diagram of injection sites in the VTA. AdipoR1^flox/flox^ + vehicle: *n* = 7 mice; AdipoR1^flox/flox^ + adiponectin: *n* = 6 mice; AdipoR1^flox/flox^/DAT^IREScre^ + vehicle: *n* = 6 mice; AdipoR1^flox/flox^/DAT^IREScre^ + Adiponectin: *n* = 7 mice. **P < 0.01, ***P < 0.001 compared with the vehicle-treated mice

To determine whether adiponectin action on firing properties of VTA dopamine neurons is mediated via activation of AdipoR1 on dopamine neurons, we examined the effects of intra-VTA infusion of adiponectin in AdipoR1^flox/flox^/DAT^IREScre^ mice in comparison with AdipoR1^flox/flox^ littermate control mice. As observed in wild-type mice, local infusion of adiponectin (0.15 μg in 0.5 μl) reduced the number of spontaneously active VTA dopamine neurons (Vehicle: 1.06 ± 0.10 neurons/track, 57 neurons from nine mice; Adiponectin: 0.54 ± 0.05 neurons/track, 29 neurons from nine mice; Wilcoxon signed-rank test, *P* = 0.008; Fig. 5c) and firing rate (Vehicle: 4.84 ± 0.23 Hz; Adiponectin: 3.61 ± 0.26 Hz; *t*_(8)_ = 14.50, *P* < 0.0001; Fig. 5d) in AdipoR1^flox/flox^ mice. However, these effects of adiponectin in the VTA were abolished in AdipoR1^flox/flox^/DAT^IREScre^ mice (population activity: Vehicle: 0.83 ± 0.11, 35 neurons from seven mice; Adiponectin: 1.07 ± 0.14 neurons/track, 45 neurons from seven mice; *t*_(6)_ = 1.876, *P* = 0.110; firing rate: Vehicle: 4.40 ± 0.46 Hz; Adiponectin: 4.29 f 0.49 Hz; *t*_(6)_ = 0.148, *P* = 0.888; Fig. 5c, d), suggesting that adiponectin acts directly on dopamine neurons via AdipoR1 to inhibit VTA dopamine neuron activity.

### AdipoR1 in dopamine neurons mediates VTA adiponectin action on anxiety-like behavior

To determine if VTA adiponectin action on anxiety-like behavior is mediated via activation of AdipoR1 on dopamine neurons, we assessed the behavioral effects of bilateral intra-VTA infusion of adiponectin in AdipoR1^flox/flox^/DAT^IREScre^ mice vs. AdipoR1^flox/flox^ control mice in the elevated plus maze test. Intra-VTA infusion of adiponectin (0.15 μg/side) in AdipoR1^flox/flox^ control mice significantly reduced the percentage of open/total arm time and entries (open/total arm time: Mann–Whitney test, *P* = 0.001; open/total arm entries: *t*_(11) =_ 3.317, *P* = 0.007; Fig. 5g). However, the effects of intra-VTA adiponectin injection in the elevated plus maze were abolished in AdipoR1^flox/flox^/DAT^IREScre^ mice (open/total arm time: *t*_(11)_ = 0.602, *P* = 0.560; open/total arm entries: *t*_(11)_ = 1.604, *P* = 0.137; Fig. 5g). These findings suggest that adiponectin acts directly on dopamine neurons to modulate anxiety-related behavior through AdipoR1 activation.

## Discussion

The results of the present study demonstrate that the adipocyte-derived hormone adiponectin can directly modulate the activity of VTA dopamine neurons and anxiety-related behavior. Our data show that adiponectin suppresses spontaneous firing activity of VTA dopamine neurons and reduces anxiety-like behavior, whereas adiponectin haploinsufficiency produces the opposite effects. Moreover, acute stress-induced activation of VTA dopamine neurons and anxiogenic response can be dampened by intra-VTA infusion of adiponectin and enhanced by targeted deletion of AdipoR1 in dopamine neurons. Further investigation revealed that AdipoR1 on dopamine neurons is required for adiponectin action on spontaneous neuronal activity and anxiety-like behavior. This is the first study to show that VTA dopamine neurons are direct targets for the action of adiponectin and mediate its effects on anxiety-related behavior, offering an explanation of the association between metabolic disorders and anxiety.

It has become well recognized that dopamine neurons in the VTA transmit not only reward signals but also signals related to aversive, stressful events [3–8]. VTA dopamine neurons exhibit heterogeneous responses to aversive stimuli [6, 7]. Stressful stimuli can either inhibit or activate dopamine neurons, depending on stress type, duration and intensity [3–7] and leading to different behavioral consequences [4, 40, 41]. These findings suggest that VTA dopamine neurons are organized into distinct functional ensembles and govern different behavioral responses. Acute restraint stress has been consistently shown to induce a pronounced activation of VTA dopamine neurons and cause anxiogenic behavior [5, 7, 40, 41]. In this study, we observed that, under basal conditions, intra-VTA infusion of adiponectin or the adiponectin mimetic AdipoRon inhibits the spontaneous activity of VTA dopamine neurons manifested as a decrease in dopamine neuron population activity and firing rate. As reported previously in rats [7, 44], we found that acute restraint stress increases dopamine neuron population activity in mice; and this effect of stress can be reversed by intra-VTA injection of AdipoRon. Behaviorally, ICV infusion of adiponectin induces anxiolytic effects in the elevated plus maze and light–dark box tests. Direct infusion of adipoRon into the VTA attenuates the restraint stress-induced anxiogenic response. These observations indicate that activation of adiponectin receptors in the VTA are sufficient to counteract the effects of stress on dopamine neuronal activity and associated anxiety behavior.

Given the reversal of the stress effects by administration of adiponectin, we asked whether an adiponectin insufficiency status would cause stress-like effects on neuronal activity and anxiety behavior. Consistent with previous reports [29, 32, 42], the heterozygous adiponectin knockout (Adipo^+/-^) mice have ~60% reduction in plasma adiponectin levels, representing a condition of insufficiency. Using this model, we were able to explore the relationships between adiponectin insufficiency, VTA dopamine neuron activity and anxiety behavior. Low adiponectin levels in Adipo^+/−^ mice led to increased firing rate of VTA dopamine neurons without a significant effect on the number of spontaneously active dopamine neurons, which differs from the effects of restraint stress exposure. This suggests that adiponectin insufficiency and restraint stress influence different firing properties of VTA dopamine neurons. However, similar to stress exposure, adiponectin insufficiency causes anxiogenic-like effects. Interestingly, increased anxiety was not observed among mice with a homozygous deletion (Adipo^-/-^) in the adiponectin gene [31, 33]. Although this non-monotonic gene dosage-behavioral phenotype relationship is at first surprising, a similar pattern of genotype/phenotype interaction has been reported in mice deficient for catechol-O-methyltransferase (COMT) and α-calcium-calmodulin kinase II [45, 46]. While we cannot completely rule out the possibility that background genes may influence this genotype-phenotype non-monotonic association, one explanation could be that compensatory mechanisms are triggered by the absence of adiponectin but not by reduced adiponectin levels. It is possible that 42% normal adiponectin levels in Adipo^+/-^ mice are below the threshold for the induction of compensatory changes. However, genetic compensation for total absence of adiponectin appears to occur for specific behavioral processes. We have recently shown that mice lacking adiponectin display impaired fear extinction [31]. Another explanation is that anxiety behavior may be differentially regulated by AdipoR1 and AdipoR2. These two receptor subtypes have distinct expression patterns in the brain and different binding affinity for adiponectin [28, 29]. AdipoR1 has relatively lower affinity for full-length adiponectin, the vast majority form [28]. In situ hybridization revealed abundant expression of AdipoR1 in the VTA, whereas AdipoR2 mRNA was almost undetectable in this region. Quantitative mRNA expression analysis by real-time PCR showed levels of AdipoR1 and AdipoR2 mRNA in the VTA were not altered in Adipo^+/-^ mice compared to wild-type littermate controls (data not shown). Thus, we speculate that a reduction in endogenous adiponectin concentrations in Adipo^+/-^ mice would cause a preferential loss of low-affinity binding sites, i.e. AdipoR1 receptor binding, which might be responsible for the anxiogenic behavior. In support of  this notion, AAV-Cre-mediated deletion of AdipoR1 in the VTA of adult mice increases anxiety, implicating dysfunction of VTA adiponectin/AdipoR1 signaling as a possible mechanism underlying anxiety-like behavior.

The physiological importance of AdipoR1 in dopamine neurons in modulating anxiety-like behavior and neuronal activity was confirmed using a conditional knockout mouse model, in which AdipoR1 was deleted specifically in dopamine neurons. Loss of AdipoR1 in dopamine neurons markedly enhanced behavioral and neuronal responses to restraint stress, suggesting that defective AdipoR1 signaling in dopamine neurons promotes susceptibility to stress. Moreover, we found that the inhibitory effects of intra-VTA injection of adiponectin on dopamine neuron population activity and firing rate were abolished in mice lacking AdipoR1 in dopamine neurons. Consistent with the electrophysiological findings, the anxiolytic effect of adiponectin was also abolished by conditional deletion of AdipoR1 in dopamine neurons. These observations suggest that AdipoR1 in dopamine neurons is essential for adiponectin action on the firing activity of these neurons and anxiety-related behavior, and that targeting VTA dopamine neurons via AdipoR1 is likely to be an underlying mechanism by which adiponectin influences anxiety behavior.

Anxiety symptoms are particularly common in those individuals with metabolic disorders [47]. It is well known that levels of adiponectin are low in people with obesity and type 2 diabetic subjects [18, 48, 49]. This study provided the first evidence that adiponectin directly target midbrain dopamine neurons, and low activation of AdipoR1 on these neurons contributes to anxiogenic behavior. Our results, in combination with previous findings [14, 15], suggest that the VTA is an important neural substrate of stress susceptibility and anxiety-related behavior that is sensitive to adipocyte hormonal signals.

## Electronic supplementary material

Supplementary Text and Figures
