# Supplementary material for: Adiponectin modulates ventral tegmental area dopamine neuron activity and anxiety-related behavior through AdipoR1
Source: Mol Psychiatry. 2018 Jul 9;24(1):126–44. doi: 10.1038/s41380-018-0102-9 (PMC6325675; doi:10.1038/s41380-018-0102-9)
Supplement: Supplementary file 1 — Supplementary Text and Figures [file 41380_2018_102_MOESM1_ESM.pdf]

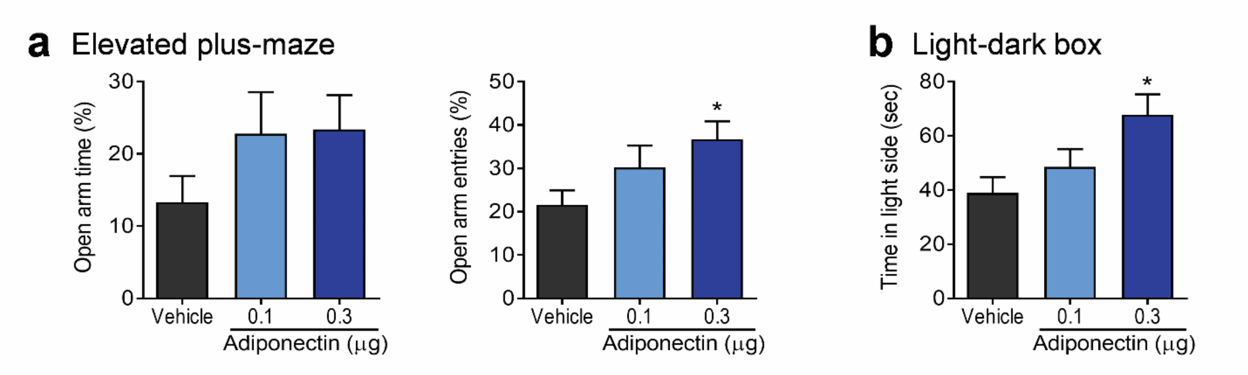

Supplementary Figure 1. Intracerebroventricular infusion of full-length adiponectin produces anxiolytic effects. Mice were implanted with guide cannula into the lateral ventricle (0.2 mm posterior, 1.1 mm lateral, and 2.7 mm ventral to the bregma). On the experimental day, a 33-gauge stainless-steel injector connected to a 5-μL syringe was inserted into the guide cannula and extended 1 mm beyond the tip. Adiponectin (0.15 μg/μL) or artificial CSF (vehicle) was infused in a volume of 2 μL over 2 min at 30 min before behavioral tests. (**a**) Elevated plus-maze test. Left, percentage of open/total arm time. Right, percentage of open/total arm entries. Vehicle: n = 18 mice; Adiponectin 0.1 μg: n = 10 mice; Adiponectin 0.3 μg: n = 14 mice. (**b**) Light-dark box test. Time spent in the light compartment. n = 8 mice per group. *$P < 0.05$ compared with the vehicle-treated group.

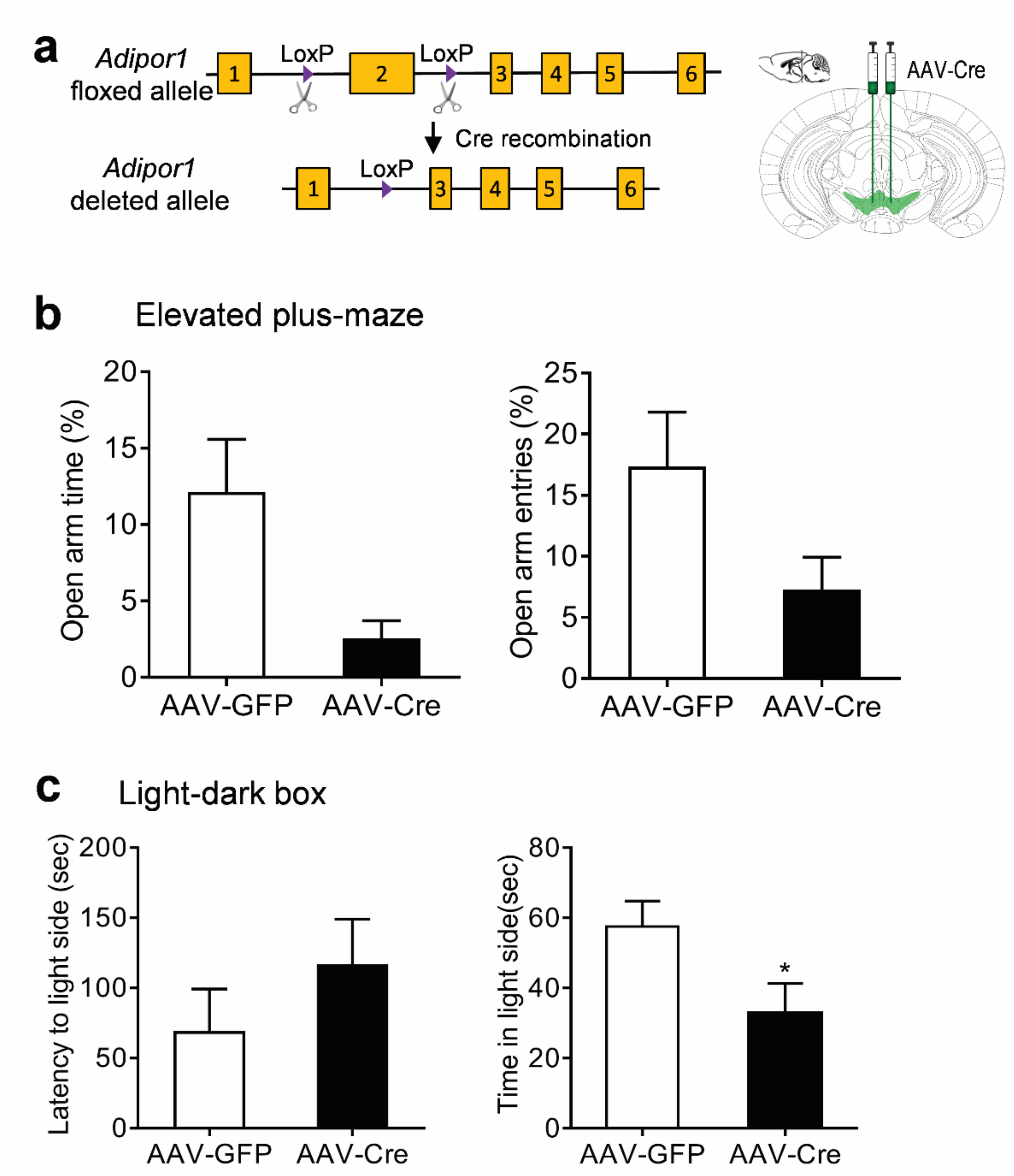

Supplementary Figure 2. Conditional deletion of AdipoR1 in the VTA. (**a**) Schematic diagram of AAV-Cre-induced deletion of AdipoR1 in the VTA. (**b**) Elevated plus-maze test. n = 7 mice per group. (**c**) Light-dark box test. AAV-GFP: n = 7 mice; AAV-Cre: n = 8 mice. *$P < 0.05$ compared with AAV-GFP injected mice.

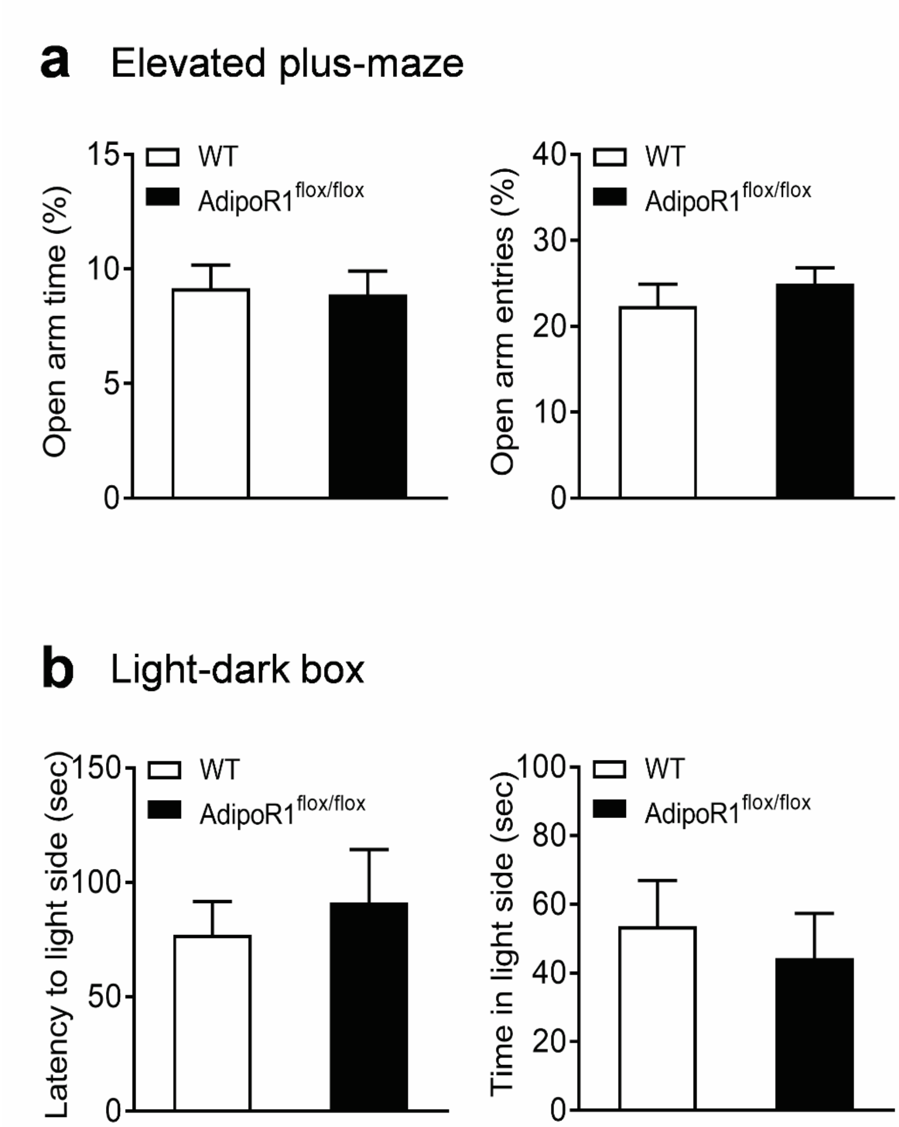

Supplementary Figure 3. Behavioral phenotype of AdipoR1$^{flox/flox}$ mice. (**a**) Elevated plus-maze test. WT: n = 7 mice, AdipoR1$^{flox/flox}$: n = 6 mice. (**b**) Light-dark box test WT: n = 8 mice, AdipoR1$^{flox/flox}$: n = 7 mice.
